# Editorial: Neuroepigenetics and biological mechanisms of stress-induced socio-cognitive changes

**DOI:** 10.3389/fnbeh.2024.1383542

**Published:** 2024-02-20

**Authors:** Willie M. U. Daniels, Duyilemi C. Ajonijebu, Olayemi J. Olajide

**Affiliations:** ^1^School of Physiology, Faculty of Health Sciences, University of the Witwatersrand, Parktown, Johannesburg, South Africa; ^2^Department of Human Physiology, School of Biomolecular and Chemical Sciences, Faculty of Science, Nelson Mandela University, Gqeberha, South Africa; ^3^Department of Psychology, Center for Studies in Behavioral Neurobiology, Concordia University, Montreal, QC, Canada

**Keywords:** stress, social homeostasis, substance abuse, depressive disorder, anxiety, neuroinflammation

Disorders of the central nervous system (CNS) have fascinated scientists and clinicians for millennia. Notwithstanding, the management and treatment of these disorders remain a huge challenge despite significant advances in our understanding of their pathophysiology. The resulting undesirable outcome for patients suffering from brain disorders necessitates ongoing research into the biological mechanisms that underpin these pathological states. A recent search on PubMed as of November 10, 2023, yielded 14,154 results when “stress-induced socio-cognitive changes” was used as the search probe. This output emphasizes the existing interest and appraised importance of understanding the topic as a major etiological factor in psychopathology. Interestingly, when the term “stress-induced socio-cognitive changes” was combined with “biological mechanisms”, the number plummets to 57 results. This significant drop highlights a critical gap in existing research and underscores the apparent need for further investigations in this area. To address this need, this Research Topic presents three full research articles and two review articles, all of which have undergone peer review and are published in this volume.

This compilation of works serves to present novel findings and updates from both preclinical and clinical studies, focusing on the biological mechanisms that may underlie three major social psychological ills namely, trauma and fear (Li et al.), stress-induced depression (Zhang et al.; Xia et al.) and substance exposure (Adeniyi et al.). The fifth study contributes by highlighting the concept of social homeostasis and presents a neurobiological framework to explain individual differences in homeostasis set points (Bales et al.). This collective effort aims to advance our understanding of the intricate biological underpinnings of CNS disorders, fostering progress in their management and treatment.

Humans inherently possess social inclinations, emphasizing the crucial role of an individual's interaction with their environment and social context in shaping their behavior. However, how this engagement is perceived varies markedly between persons. Bales et al. recently postulated that each individual possesses a distinctive social homeostasis setpoint, influenced by information received through a detector system. This information is then processed by a control center, which determines the output via an effector system. The outcome is the individual's ability to assess the potential consequences of a social context, enabling them to make decisions regarding whether to associate or avoid such social engagement. Disruptions in establishing this social homeostasis may therefore trigger abnormal mental states.

The global increase in life stress, wars, social violence, natural disasters and fatal accidents has seen an escalation in the exposure to traumatic events. Witnessing such phenomena often results in a heightened consolidation of the memory of the adverse event and subsequent difficulty in extinguishing such memories. Trauma-induced fear in this case is underpinned by abnormal memory formation. Consequently, gaining insights into the overarching macro (between brain regions) and microcircuitry (within a brain region), along with the molecular mechanisms, provides a promising platform to effectively address fear-related disorders. This perspective is underscored in the study by Li et al..

The interest in studying biological mechanisms alternative to neuroanatomy, neurochemistry, and neuroendocrinology, has shifted to psychoneuroimmunology. The earlier doctrine that the brain is protected against immunological onslaughts has been revised following the emergence of evidence showing an active central immune response in some disorders of the brain (e.g., HIV associated neurodegenerative disorder, Alzheimer's disease). The important role of the neuroimmune system in precipitating pathology has now been fully embraced and its contribution to the development of CNS disorders has become an active area of research. The role of psychoneuroimmunological mechanisms in major depression is one such area. In particular, the scientific grounds for the disparity in the prevalence of major depression between different sexes, have captivated many scientists. A rodent-based study investigating the role of cytokines in this regard, provides some insight into this matter (Xia et al.). Moreover, a study by Zhang et al. underscores that prenatal exposure to inflammation heightens the risk of offspring to neuropsychiatric disorders such as anxiety, depression, and cognitive dysfunction, while maternal sleep deprivation during pregnancy exacerbates these outcomes. The authors further observed that the behavioral and cognitive impairments are intricately linked to changes in immune responses, marked by increased expression of proinflammatory cytokines.

Another huge social ill that has a profound negative impact on communities is substance abuse. The prevalence of substance use and abuse continues to escalate globally at an alarming rate. Of particular concern is the rising prevalence of polydrug use and the consumption of clandestine-produced street drugs, as highlighted in the UNODC World Drug Report (2022). Despite tremendous efforts by law-enforcement agencies drug trafficking remains rife, while rehabilitation and treatment centers battle to manage substance users effectively. One reason for this battle is the current incomplete understanding of the pathophysiology of substance use disorder (SUD). While massive strides have been made regarding the neurocircuitry, brain regions and neurotransmitters involved in the manifestation of SUD, the biological mechanisms underlying intergenerational transfer of susceptibility and the impact of prenatal drug exposure are still to be fully elucidated. Here, preclinical studies have proven to be useful in offering some explanation of the phenomena. As such a focus on alterations in the expression levels of relevant genes is presented (Adeniyi et al.).

In summary, this Research Topic compiles a series of articles and an innovative framework that illuminate the complex dynamics of social homeostasis, susceptibility to anxiety or fear-related disorders and stress-induced depression. Additionally, it addresses the escalating global concern of substance abuse and the incomplete understanding of the pathophysiology of SUD, underscoring the potential insights gained through preclinical study that focus on alterations in gene expression.

## Author contributions

WD: Project administration, Supervision, Validation, Writing—original draft, Writing—review & editing. DA: Conceptualization, Project administration, Supervision, Validation, Writing—review & editing. OO: Project administration, Supervision, Validation, Writing—review & editing.

